# Historical Perspective: The social determinants of disease – some roots of the movement

**DOI:** 10.1186/1742-5573-2-2

**Published:** 2005-04-19

**Authors:** S Leonard Syme

**Affiliations:** 1 University of California, Berkeley, School of Public Health 140 Warren Hall, Berkeley, CA 94720 USA

## Abstract

This is an account of the early days of research on social determinants as I experienced them. I describe my time as one of four Fellows in a new training program in Medical Sociology at Yale University and how I came to be the first Sociologist employed in the U.S. Public Health Service. I then became the first Executive Secretary of a new Study Section at NIH dealing with a small number of research grant proposals in the field of Epidemiology. My account deals with some of my experiences in this developing field, culminating with my appointment as the first Sociologist to become a Professor of Epidemiology in a School of Public Health.

## Introduction

In 2001, a colleague and I wrote an article describing a remarkable phenomenon: between the years 1995 and 2001, there had been ten books published that focused on the social determinants of disease [1]. We suggested that this explosion of work marked the coming of age of the field of social epidemiology. Since that paper appeared, more than a dozen new books dealing with Social Epidemiology have been published, more than a dozen Reports from the Institute of Medicine (National Research Council) have been written on such topics as social and behavioral approaches to racial and ethnic inequalities in health, and hundreds of journal articles have been written on these issues.

In addition to these publications and reports, the Robert Wood Johnson Foundation has recently established a new program to train postdoctoral scholars in a field they refer to as Health and Society; this training program has a heavy emphasis on the study of the social determinants of disease and is, I believe, the first major national initiative ever taken to train students in social epidemiology. Perhaps in response to these developments, I was asked by the Department of Epidemiology at the Mailman School of Public Health at Columbia University to give a lecture on "The Social Determinants of Disease: The Roots of the Movement". They wanted me to describe the "beginnings" of the field of social epidemiology on the basis of my personal experience. I received many comments following that talk suggesting it might be of interest to record my remarks in published form and that is the purpose of the present paper.

It is of course presumptuous for me to discuss the beginnings of this work based on my own limited exposure. The real beginning probably begins with Hippocrates and includes such other early scholars as Louis Villerme, Rudolph Virchow, Edgar Sydenstricker, and Emile Durkheim as well as more recent scholars such as Thomas McKeown, Saxon Graham, Mervyn Susser, Leo Reeder, Bruce Dohrenwend, Sol Levine, and John Cassel. My limited personal experience should therefore be considered within this very much broader context.

## Analysis

My first attempt to think about my beginnings of work in social epidemiology was described in the Foreword to the Berkman/Kawachi textbook on Social Epidemiology [2]. As I indicated in that Foreword, my thinking about social determinants began in 1955 when I was accepted into a training program in the Department of Sociology at Yale University. That training program, called "medical sociology" was funded by the Commonwealth Fund and it was the first such formal training program in the world. There were four of us in the program. We were given a choice early on as to whether we would focus on what was then called the sociology *of *medicine or sociology *in *medicine. As I noted in the textbook, the logical choice was for me to choose to study the sociology *of *medicine because there already existed a relatively large and interesting literature on this topic dealing with the institution of medicine and medical care, the sick role, and attitudes and beliefs of patients regarding illness, pain, and medical treatment.

For reasons that are not clear to me, I decided to study sociology *in *medicine, which I took to mean the study of how social factors affect health and well-being. I now realize that what my Professors at Yale really meant by this term was nowhere near as grandiose as my version. Professors August Hollingshead and Frederick Redlich were at that time doing a large study of the link between social class and mental illness and that is what they meant by the term "sociology *in *medicine": They wanted me to help them with their research. I had no interest in the topic of mental illness because I took it for granted that social factors would somehow be related to mental illness. Looking back, I can see what a naïve view this was, but that was my uninformed position at that time. Instead, I wanted to know if social factors were related to diseases that were *not *so obviously connected to the social world, diseases such as heart disease, cancer and arthritis. Not only was this a naïve view, but it was also a reckless decision because there was virtually no literature on these topics at the time and no one was sure there ever would be.

When I graduated, I was scheduled to go into the Army to fight in Korea but Professor Hollingshead said that I might want to consider an alternative that would give me a military deferment: go to work for the U.S. Public Health Service in Washington. I agreed that that was a better idea. He said that he recently had talked to a statistician in the Heart Disease Control Program in Washington who wanted to hire a sociologist. So I went to Washington and met Phillip Enterline. I asked him why he wanted to hire a sociologist to study heart disease and he said that he had no idea. He and his group had just completed a study of the geographic distribution of coronary heart disease mortality in the U.S. and they found very high rates on the East and West Coasts and in the Detroit-Chicago metropolitan area but low rates elsewhere. They had not been able to explain this finding and they thought that perhaps a sociologist might be able to help.

So I took the job. I was to be classified as a Statistician in the Civil Service because there was no category available for a Sociologist. I made the mistake of reporting this to Professor Hollingshead and he was not very happy. "If there's no category for a Sociologist, make one!" He raised such a fuss that they in fact did. So I was the first Sociologist labeled as such in the Civil Service.

Then I went to work and it was a disaster. I decided to begin my work by looking at data from a state with a very low death rate from CHD with the idea of then doing a similar study in a higher rate state. We obtained some wonderful data from North Dakota, a low rate State. In a six-county area of North Dakota, we were able to obtain information on every case of coronary heart disease that occurred in men, 35–64 years of age, in a one year period. Then we selected two age-matched men, free of CHD, from a representative sample of the 6 county area from which the cases came. I then set about testing all the hypotheses that I had learned in graduate school. In those days, we were thinking about marginality, status crystallization and many other concepts that no one can now remember. It must be recognized, of course, that there was no literature or previous research to rely on. This was, I think, the first such study of CHD ever done with social factors. So I based my work on the concepts that I had been studying in school. I spent a year doing this. Not one of the hypotheses worked out. The cases and controls did not differ from one another on any of the dozens and dozens of ideas that were then popular in Sociology. I think Enterline must have thought he made a major mistake in hiring me.

So I decided on a different tack. I would go through all of the data and see on which items there might be a difference between cases and controls. I had been taught that this type of fishing expedition was not a very good way to proceed, but I was desperate. In this analysis, I was able to see a considerably higher rate of CHD among men who had changed jobs and who had moved geographically and, especially, among men who had moved from farms to white collar jobs in the city [3]. I observed all of this, of course, after controlling for smoking, blood pressure, and many other CHD risk factors. I called this phenomenon "cultural mobility" [4]. I was then able to repeat this analysis with a remarkably similar data set in a State with a much higher rate of CHD, California [4]. And I found precisely the same thing as in North Dakota.

I came to a fateful decision based on this experience. I decided that our social theory was not very useful in helping us think about health matters. I decided to no longer base my research on theory but to collect reasonable seeming data and do fishing expeditions. I taught several generations of students to forget the "theory thing" and just go for it. The result is that we now in social epidemiology have piles and piles of findings and no way to make sense of it or to think about what needs to be done next. This sorry situation is not all my fault of course but I have been a major contributor. And the reason for it is to be found in the wheat fields of North Dakota. Fortunately, better minds than mine are now prevailing and things are getting better. For example, one of my former students, Nancy Krieger, is forcefully demonstrating the power, and importance, of theory in spite of everything I tried to teach her [5]. This part of my work has not been one of my better contributions.

I was prevailed upon by Professor Jeremiah Stamler to present my North Dakota findings at a meeting of the American Heart Association. It was a daunting experience. In the front row sat all of the most eminent cardiovascular epidemiologists in the world and I suggested that above and beyond the usual CHD risk factors was a set of social factors that no one could understand and for which possible disease mechanisms were very difficult to visualize. One eminent epidemiologist cornered me after my presentation and angrily criticized the whole approach. His argument: "What are we supposed to do with findings like this? Tell people not to move or change jobs? All you are doing is distracting people from the real issues which are cholesterol, blood pressure and smoking. You are doing shameful work and you should stop it!"

I did not stop, of course, but these were difficult times. I had other troubles on other fronts. For example, a nutritionist on the staff of the Heart Disease Control Program asked me to help her design a dietary questionnaire. These were the days before we had the well-established instruments we have today. She wanted to do a study of Seventh Day Adventists. There was at that time, 1959, a growing body of evidence, and speculation, that a diet high in fat might be a risk factor for coronary heart disease. Seventh day Adventists were lacto-ovo-vegetarians and it was thought to be interesting to study their lipid levels and other health issues. I agreed to help her design a questionnaire. As we worked, it occurred to me that Adventists might have better lipid levels not only because of their diet but because they were religious. So I convinced her to let me add three questions at the end of the interview about their church attendance and about the importance of religion in their lives.

Since this was a government survey, all forms had to be cleared by a group in the Bureau of the Budget. Two weeks after we submitted our questionnaire, word came that it had been approved but that my three questions on religion had been deleted. I was not very happy. Upon inquiry, I was informed that there is in the U.S. Constitution a policy of separating church and state and that my three questions, on a government form, violated the Constitution. So I handed in my resignation. An Assistant Surgeon General summoned me to his office the next day. "What's all this about quitting?" he asked. I told him that as a sociologist I needed to ask people questions about their lives, including their religious beliefs, and if I wasn't going to be able to do that, there was no point in my working in the government. He told me to calm down. He asked if there was any evidence to support my hypothesis that religious beliefs had anything to do with lipid levels. "Of course there is!" I lied. "That's why I put those questions in!" "OK," he said, "bring me the evidence and then we'll talk". I went to poor suffering Phil Enterline and asked him for 3 weeks off so that I could search for the evidence that I had so confidently said existed.

I worked very hard during those three weeks and I did in fact find quite a bit of evidence. There was information about religion and stress taken from studies of Trappist and Bendictine monks and there was evidence about stress and lipids from studies of medical students at exam time and from tax accountants at tax time. I also did a lot of research about the Seventh-day Adventist religion and its relevance for stress research. As a complete amateur, I concluded that the SDA religion was based on the return of the Lord and that that return will occur soon after we see people warring with one another and when there is much civil strife and, in general, when everything is falling apart. So I argued that Seventh-day Adventists have a very different response than the rest of us when they read the daily newspaper. We moan about the events of the day while they see the bad news as bringing them closer to salvation.

I wrote a 45 page paper about stress and lipids, religion and stress, and about the Seventh-day Adventist religion. It was, I must say, quite elegant. I ended with a paragraph saying that in light of the foregoing, the three questions I wanted to ask were clearly warranted. I handed in my paper and was summoned a few days later to the Assistant Surgeon General's office. He said he was impressed with my paper and that he was satisfied that there was a credible scientific basis for my three questions. "But," he said, "we now have to consider the constitutional issue". I felt betrayed and said I was going to resign. Again, he told me calm down. "Give me a few weeks", he said. Several months later, he announced a change in government policy about such issues. One can now ask about things like religion if a case can be made that more good than harm will come from the inquiry. There has to be a good, or even compelling, reason for violating the constitution, but it can be done.

A year later, in 1960, I was asked to move to the National Institutes of Health to establish, for the first time, an Epidemiology Study Section. This was a very powerful position for a young 28-year-old beginner. This new Study Section was to be established to deal with the small number of research grant applications that were beginning to be submitted to the NIH dealing with the epidemiology of such non-infectious diseases as arthritis, mental illness, cancer, heart disease, and injuries. I had received my Ph.D. in a new field called Medical Sociology just three years prior to this invitation and, while I had been working as a fledgling epidemiologist in a heart disease program in the U.S. Public Health Service, I was not very knowledgeable about the field; not many others were either. My boss at NIH, Dr. Murray Goldstein (who later became the Director of the National Institute of Neurological Disorders and Stroke) asked me to nominate a group of people who could serve on this new Study Section and I began to do research to deal with this challenge. The first thing I learned was that we could not use the word "Epidemiology" for the title of the new Study Section because that word was reserved for the study of infectious diseases only. We therefore came up with an alternative name, the "Human Ecology Study Section". We then selected a truly distinguished multi-disciplinary group of members.

Our first choice was Abraham Lilienfeld from Johns Hopkins University. Even then, he was the outstanding epidemiologist in the country. Then there was William Cochran from Harvard, perhaps the most outstanding biostatistician in the country. And Arno Motulsky the geneticist then at Washington University. Other members included John Fulton (a dentist), William Clark (an infectious disease epidemiologist), Schulyer Kohl (an obstetrician), Felix Moore (a biostatistician), George Reader (an internist) and Robert Shank (an internist and nutritionist). And, because of my training as a sociologist, I nominated my Professor from Yale, August B. Hollingshead (who, as I noted earlier, was beginning to do pioneering work on the link between social class and mental health) and Otis Dudley Duncan, from Chicago, who was working on the relationship between macro social forces and behavior. There were no women or minorities on the Committee reflecting the fact that there were very few women and scholars from minority groups working in this area at that time.

The Human Ecology Study Section, later renamed the Epidemiology Study Section, eventually grew into several large subdivisions. In those days, however, there were very few applications to review and we took it as our mission to help develop the field. For that reason, we went on site visits very frequently. If a grant proposal looked promising, but inadequate, we went to visit the group to help them do it better. I was on airplanes all the time. It was a truly fascinating experience. We visited John Cassel in North Carolina. He was doing some of the very best work at the time and, interestingly, much of his research is still the best. He was doing a study about the health consequences of people moving from rural places to take jobs in factories. We went out into the hills of western North Carolina to visit a paper mill that was one of his factory sites. We met a remarkable young occupational physician who we later induced to come to Chapel Hill to study Public Health. That was the beginning of Herman (Al) Tyroler's distinguished career in Epidemiology. We gave a young Warren Winkelstein his first grant to study the health effects of air pollution in Buffalo, New York. We supported Lawrence Hinkle's work on stress in telephone workers. We supported research on Seventh Day Adventists to see if their good health was due to nutrition or spirituality. We supported Sam Shapiro's pioneering study of mammography in HIP. We supported Saxon Graham who was studying the link between social factors and cancer at Roswell Park. We supported Bruce Dohrenwend's classic work on mental health. And we supported the work of Sol Levine and Norman Scotch in their study of social factors in the Framingham study. There was at that time a lot of money available and we were able to work hard to stimulate epidemiologic research. Since I was the Executive Secretary and trained in Medical Sociology, quite a lot of that support went to beginning work in social epidemiology.

I recall a time during those years when Dr. Lester Breslow applied to the NIH for money to support the establishment of what he called a Human Population Laboratory in Alameda County, California. His idea was to do research in a large representative sample of an entire county over a long period of time to study what he called their health in relation to their way of living. What disease was he going to focus on? None. He had been influenced by the writings of John Cassel and others suggesting that an appropriate outcome for studies of social factors might be "health and disease" in general and not one or another specific disease. This idea was later eloquently presented in the last paper Cassel wrote before he died. I am referring to his classic contribution published in 1976 in the American Journal of Epidemiology called "The Contribution of the Social Environment to Host Resistance" [6].

The NIH was not sure how to deal with Dr. Breslow's proposal because it did not neatly fit into any of the disease-specific institutes. It turned out that there was no institute at the National Institutes of Health that dealt with health. Of course, this is still the case. I was asked for my advice on how to handle this very unusual application. I suggested that we develop a special study section with specially chosen people to deal with this crisis and I was given permission to proceed. To create this special review committee, I invited a few people from my Study Section to serve, as well as some carefully chosen outsiders. I attempted to pick people who I thought could understand the radical idea that Dr. Breslow was proposing.

We went out to California for a two-day meeting. In the end, my specially picked people recommended that the proposal not be funded. It was too weird. For example, Breslow proposed to study the health of people but he was not going to do one physical exam or take any blood or urine. He was simply going to ask people to rate their own health! I recall he proposed a question that asked "Compared to other people your age, how would you rate your health? Excellent, good, fair, or poor?" This question has turned out to be one of the most powerful predictors of future health in dozens and dozens of studies but at that time it was a very bizarre question indeed. That the Breslow proposal was turned down was very disappointing but I urged Dr. Breslow to resubmit and a year later I assembled yet another group of specially picked reviewers to give it another try. And this time it worked. So Dr. Breslow was able to establish this crucial population study that has turned out to be one of the most significant studies in the history of social determinants [7]. A few years later, Dr. Tommy Francis of the School of Public Health in Ann Arbor was able to establish a similar population laboratory in Tecumseh, Michigan.

After three years of serving as Executive Secretary, I returned to do research in the Heart Disease Control Program, this time based in San Francisco, California. By then, it was clear that a field of research in social epidemiology was emerging, most of it focused on coronary heart disease. This research was not of very high quality, nor were the results compelling, but some interesting questions were beginning to emerge. I had a conversation with Professor Leo Reeder about this and we decided it might be good to bring together all the people who were engaged in this research to see what we were all doing and to think about next steps. Since I was a government employee, I prevailed upon my bosses to provide funds for the meeting. The Conference was held in Phoenix, Arizona in February 1966. We invited all of the social scientists and medical people in the country doing research on heart disease as well as some others who, while they were not doing such research, were nevertheless bright and potentially helpful. We scoured the country and came up with 27 people, including Reeder and myself.

The report of our conference was later published in 1967 as a special volume of the Milbank Memorial Quarterly with the title "Social Stress and Cardiovascular Disease" [8]. It is a little embarrassing to read the book now and see the state of the art at that time but it was quite clear that, in spite of this, something important was happening. It was in one of the last papers of this little book, by the way, that I explored the issue of appropriate outcomes for social epidemiologic research. I argued in that piece, no doubt influenced by Lester Breslow's idea for the Alameda County Study, that we needed to look at a broader set of disease outcomes than the usual clinical entities. This idea stands as one of the key features in John Cassel's classic paper as well.

In 1968, I became a Professor of Epidemiology in the School of Public Health at Berkeley. I was, I think, the first sociologist to hold a position as an epidemiologist at any School of Public Health in the world. Leo Reeder was a sociologist at the UCLA School of Public Health but his was a normal position as a Professor of Behavioral Science. By then I was working with Reuell Stallones, who was also a Professor at Berkeley, to study coronary heart disease and stroke among Japanese migrants to Hawaii and California. Stallones was primarily interested in testing the dietary hypothesis. Did the Japanese in Japan have low rates of CHD due to their low fat diet? I was interested in testing the mobility hypothesis. Did rates of CHD go up among the migrants? We were both surprised by the findings.

It turned out that Japanese men who migrated to California had CHD rates five times higher than those in Japan, with migrants to Hawaii having intermediate rates. And this increase in CHD rate was not explained by any of the usual CHD risk factors such as diet, serum cholesterol, smoking or blood pressure. I assigned a doctoral student to figure out what was going on. Michael Marmot did his doctoral dissertation on this issue. He concluded that those Japanese men who had adopted Western cultural ways were the ones with the enormous increase in CHD while those California Japanese who had retained traditional ways had rates comparable to those still living in Japan [9]. Again, this observation was independent of diet and all the usual CHD risk factors. This clearly was not supportive of the mobility hypothesis since some migrants had no health consequence at all. Then Marmot left Berkeley to go to London to begin his work on the British civil servants and he left me with the question: what does it mean to say "Western ways" versus "Traditional ways"?

I went to Japan several times, interviewed dozen and dozens of people, and I read many books to get some understanding of this but all I could get out of this work was that my Japanese informants thought that Americans were lonely. I challenged this observation many times but dozens of people said that anyone could easily see this loneliness when you saw so many Americans walking on the street, alone. "Alone on the street?" I said. "That's not evidence of loneliness", I said. People all shrugged at my naiveté. So I returned to Berkeley, this was in 1975, and found another doctoral student who agreed to work on this. That student, Lisa Berkman, had already been thinking about the importance of social networks and social support  and took took my loose and primitive question and reshaped it into a brilliant and elegant study showing the health consequences of social connection. For this study, she used data from the Alameda County Human Population Laboratory that Lester Breslow and I had worked so hard to get funded many years before. My view is that Berkman's study, published in the American Journal of Epidemiology in 1979, really began to establish the field of social determinants [10]. Her findings were later replicated by James House and his group using data from the Tecumseh study [11]. This was a finding that resonated with common experience and that fit with many of the empirical observations we had been making over the years. And it was entirely consistent with one of the most important contributions ever made in social epidemiology: the work of Emile Durkheim on Suicide [12]. If one is going to talk about the roots of a movement, it is crucial to put Berkman's and Durkheim's work at the center.

In my classes at Berkeley, I provoke all first year students by assigning them to read a significant number of pages from Durkheim's work on suicide. Physicians are especially challenged. Here, in suicide, is a study of one of the most intimate and personal behaviors that can be imagined. Surely, Durkheim notes, this behavior can only be explained by understanding the most intimate personal events in someone's life. And yet, he points out, there is a patterned regularity in suicide rates, over time, in various groups. Some groups have characteristically high or low rates of suicide, over time, even as individuals come and go from these groups. If the causes of suicide are to be found within the individual, he asks, how can there be a patterned regularity in groups over time even as individuals come and go from these groups? There must be something about the groups themselves that causes a higher or lower rate. That something would not explain why only some individuals succumb to the social fact but it would explain the difference in group rates. A better description of the role of social epidemiology does not exist. Berkman's work on social connections was the first modern empirical demonstration of Durkheim's genius. And since her work, of course, the importance of social networks has become a recognized international fact. And it has led me, and others, to think about such concepts as control and other similar factors that might explain inequalities in disease by social class.

The Alameda County Human Population Laboratory has been useful for other important work as well. George Kaplan and Mary Haan used data from this study to do their pioneering work showing that certain neighborhoods had higher and lower rates because of their poverty status and to show that these differences could not be explained by the characteristics of individuals living in those areas [13]. Another Durkheim legacy. Others who worked with this data set were Jack Guralnik, now at the National Institute of Aging, John Lynch, now at the University of Michigan, and Teresa Seeman, now at UCLA. And most recently, Irene Yen. And there were others. The special Study Section authorized by the NIH clearly made an important contribution. And the findings obtained from the Tecumseh Human Population Laboratory, another legacy of the Study Section, has also been impressive.

This is a very sketchy and highly selective personal set of observations about the early years of the movement as I experienced it. I have left out a lot and I have undoubtedly ignored the work of many others at work at that time. The result of all of these efforts, however, is revealed today not only by the appearance of the new books mentioned earlier and by the Robert Wood Johnson program but also by the fact that both the National Institutes of Health and the Centers for Disease Control are emphasizing work in this area under the rubric of "disparities". In addition, the Canadian government has reorganized its grant-giving mechanisms to recognize this work by establishing a new Institute of Population and Public Health. To me, it is amazing to see the changes that have occurred in the last 40 or 50 years.

What is the explanation for this phenomenon? In my experience, these changes have not come easily. In fact, my experience has been that they have come very grudgingly, with great suspicion and wariness. This suspicion, it has seemed to me, was based on the following issues: First, many felt that social determinants were vague, and ill-defined concepts based on poor (that is non-experimental) research. Second, even if research findings were shown to be well documented, it was very difficult to imagine how these social factors could "get into the body" to cause disease. Third, even if associations between social factors and disease were well documented and even if a disease mechanism could be imagined, it was difficult, if not impossible, to see how these factors could be intervened upon. Current work on social determinants is focused on these very issues and with very promising results.

## Conclusion

As I look back over the last 50 years, I am enormously impressed, and a little surprised, at the positive changes that have taken place in our work to improve health and well-being. These changes began very slowly many years ago by a relatively small group of people but the pace of change is increasing exponentially. Because it is the right thing to do. And because we really have no choice: We all know that our medical care system is under enormous strain. And we all know that the baby-boomers will enter the over age 65 group between 2020 and 2030. When they do, the number of old people in our country will have doubled. If we think the medical care system is under stress now, we will soon see the system burdened even more dramatically. Our only hope is to develop better programs to prevent disease in the first place and not merely wait to fix things after the fact. And to develop appropriate programs of prevention, we are going to need vital, vigorous and creative research and intervention activities that are firmly rooted in social epidemiology. It would be fun to look back 50 years from now to see how all this works out.
